# Comparing genetic differentiation and variation using ISSR and SCoT among Juniper plant markers in Saudi Arabia

**DOI:** 10.3389/fpls.2024.1356917

**Published:** 2024-03-22

**Authors:** Hatim M. Al-Yasi, Rahmah Al-Qthanin

**Affiliations:** ^1^Department of Biology, College of Science, Taif University, Taif, Saudi Arabia; ^2^Biology Department, College of Sciences, King Khalid University, Abha, Saudi Arabia

**Keywords:** genotypes, *Juniperus procera*, polymorphism, principal coordinate, Saudi Arabia

## Abstract

Juniperus, a genus of fragrant evergreen trees in the Cupressaceae family, encompasses up to 67 distinct species distributed globally. Among these, *Juniperus procera*, also known as the East African pencil cedar or African Juniper, stands out for its notable medicinal properties. Due to the well-recognized therapeutic benefits of Juniper species, assessing genetic diversity is essential for various breeding initiatives. Thus, in this work, six ISSR and six SCoT primers were utilized to evaluate the genetic diversity between 23 *Juniper* genotypes collected from different locations in Saudi Arabia. 29 out of 103 and 26 out of 105 amplified bands, respectively, were found to be polymorphic markers using the ISSR and SCoT studies. With the help of 120 genotype-specific markers, including 60 for ISSR and 60 for SCoT, several varieties of *Juniper* were discovered. In addition, the polymorphism information content (PIC) was computed to assess the effectiveness of the markers. The findings of this study highlight the importance of conserving the genetic diversity of *Juniperus procera*, as it holds immense potential for developing new medicinal products. Additionally, the results provide valuable insights into the genetic structure of Juniper populations in western Saudi Arabia, which can inform future conservation and management efforts. However, all of the techniques utilized to profile the genotypes of Juniper can be regarded as useful techniques for long-term fingerprinting and diagnostic markers.

## 1 Introduction

*Juniperus procera* (commonly called Arar in Arabic) is an important medicinal plant in the family Cupressaceae (Hazubska-Przybył, 2019). Due to the diverse medical characteristics associated with the *Juniperus* genus, junipers (*Juniperus* spp.) are significant pharmaceutical plants and are frequently planted throughout the northern hemisphere; nevertheless, despite the fact that they are important for both industrial and pharmacological purposes and despite the fact that professional breeding programs frequently research plant diversity. With more than 50 known species and 36 variations worldwide, the *Juniperus* genus is a significant member of the Cupressaceae family (Salih et al., 2021). Correspondingly, in the province of Taif, only *J. procera* and *J. phoniceae* are present; these two juniper varieties coexist at elevations ranging from 1700 to 3000 meters above sea level, with *J. phoniceae* prevailing at higher altitudes of around 1700 meters in the northern part of the Sarawat Mountains, and *J. procera* dominating in the southern half. In the southern region of Saudi Arabia, Juniperus is widely distributed, particularly in the Asir Mountains, with some mixed populations near Taif. In flat areas, these trees can grow to heights of 10-15 meters, but on slopes, they only reach a few meters in height (AL-Ghamdi and Jais, 2013). The pharmaceutical value of juniper plants stems from the presence of various specialized compounds, including flavonoids, lignans, coumarins, sterols, and terpenoids, which are thought to be a source of natural medications that may have antifungal, antioxidant, insecticidal, anticancer, and antibacterial properties (Ghorbanzadeh et al., 2021; Salih et al., 2021). The species has been steadily disappearing worldwide, primarily due to drought, soil erosion, temperature changes, and increased runoff; in several nations, it is classified as an endangered tree (Abrha et al., 2018). Due to these natural fluctuations, populations of *J. procera* exhibit various patterns of genetic and chemical variation in different geographic locations (Chen et al., 2015). However, molecular markers have demonstrated a significant role in the ecological restoration process to understand the species; the molecular variation of plants can be associated with specific chemical phenotypes and is primarily responsible for their ability to adapt to new environmental conditions (Pacheco-Hernández et al., 2021).

Genetic diversity studies for a few *J. procera* species have been linked to molecular markers such as start codon targeted (SCoT) and inter simple sequence repeat (ISSR) (Alsamman et al., 2019 and Alzahrani et al., 2023). These molecular marker techniques are widely used because they are crucial and may produce an enormous quantity of DNA markers in every test to investigate genetic variations. It is essential to experiment with hardware, look into funding, and select appropriate marker tactics that align with the plant species (Alotaibi and Abd-Elgawad, 2022).

ISSR markers are loci linking specific genetic information and duplicate DNA fragments between two identical microsatellite sites (Zargar et al., 2023). ISSR is highly polymorphic and essential in research on developmental, hereditary processes, biodiversity, and genome mapping; this PCR-based method can address some of the drawbacks of existing marker techniques, including the expensive nature of AFLP and the poor reproducibility of RAPD, and is applied to a wide variety of plant species (Alotaibi and Abd-Elgawad, 2022). In this sense, the SCoT marker, which directs the coding region of the plant genome by focusing on the sequence around the ATG codon, has gained increasing popularity and is considered one of the critical molecular markers Jedrzejczyk (2020); it has proven to be more efficient compared to other random markers caused of their high annealing temperatures and longer primer lengths and designing analyses of it does not necessitate extensive knowledge of the genome sequence (Abulela et al., 2022). In order to ascertain the genetic diversity of *J. procera* (Arar) plants from various Saudi Arabian locations, ISSR and SCoT markers were used. As a result, the findings of this study will be helpful in understanding how plant genetic variation and plant breeding interact.

## 2 Materials and methods

### 2.1 Study area

**Figure 1:**
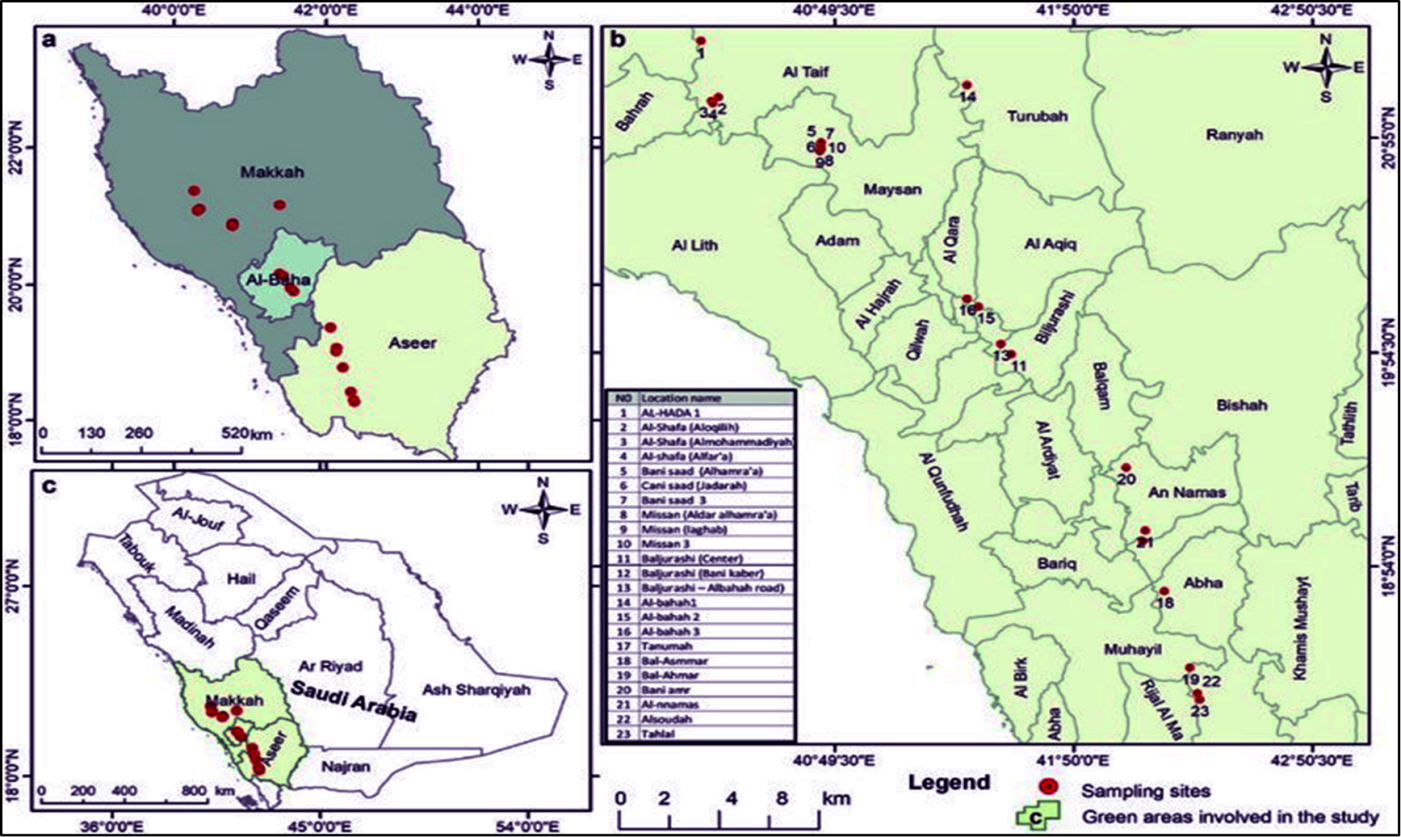
Location, Geographical coordinates, and Altitudes of the twenty-three cultivars of *J. procera*.

In 2022, twenty-three *J. procera* plants were gathered from their natural habitats in the Southwest region of Saudi Arabia. Accessible in (**Figure 1**) is the geographic distribution of gathered accessions and their sites of interest. The source of DNA synthesis was the leaves of plants.

### 2.2 DNA extraction and quantification

DNA was extracted from *J. procera* leaves utilizing an Aquadien extraction Kit (Bio-Rad, Cat. No. 1665007, USA) following the manufacturer’s instructions. The amount and purity of DNA in samples were calculated using a NanoDrop 2000 Spectrophotometer (Thermo Scientific, Germany).

### 2.3 ISSR amplification

The PCR reaction was conducted using the Thermo Fisher Scientific apparatus (Applied Biosystems, USA) and involved six ISSR primers and six SCoT primers (**Table 1**). A final volume of 20 μl was used for each reaction, comprising 2X PCR master mix (OnePCR™ GeneDireX, Cat. No. MB203-0100, Taiwan), 2 μl of template DNA (around 50 ng/μl), 2 μM/μl of forward and reverse primers, and finally nuclease-free water. The amplification process commenced with a 5-minute annealing period at 94°C. Subsequently, 35 cycles were performed, involving 1 minute of denaturation at 94°C, primer annealing for 1 minute at 45°C for ISSR primers and 50°C for SCoT primers, and primer elongation at 72°C for 2 minutes. The final step consisted of 5 minutes at 72°C (Guo et al., 2012). Finally, the products were separated using a 2% agarose gel.

**Table 1:** The nucleotide sequences of ISSR and ScoT primers.

Primer Name	Sequence (5′–3′)
ISSR-1	AGAGAGAGAGAGAGAGYC
ISSR-2	ACACACACACACACACYA
ISSR-3	ACACACACACACACACYG
ISSR-4	ACACACACACACACACG
ISSR-5	ACACACACACACACACYT
ISSR-6	ATACACACACACACACAT
SCoT-1	ACGACATGGCGACCACGC
SCoT-2	AACCATGGCTACCACCAC
SCoT-3	ACGACATGGCGACCATCG
SCoT-4	CCATGGCTACCACCGCCT
SCoT-5	ACCATGGCTACCACCGCC
SCoT-6	ACCATGGCTACCACCGCA

### 2.4 Data analysis

Since only distinct and unmistakable bands could be visually scored for all reactions, and since the final data sets contained both polymorphism and monomorphic bands, the binary matrix was developed based on the presence (1) or absence (0) of DNA fragments. The unweighted pair group technique with arithmetic averages (UPGMA) was then used to determine the genotype-to-genotype similarity matrix coefficients. Correspondingly, using the PAST program Version 1.91, principal coordinate analysis (PCA) was then carried out using the Euclidean similarity index on this matrix to create a phylogenetic tree (dendrogram) (Hammer and Harper, 2001). Also, iMEC (https://irscope.shinyapps.io/iMEC/), we used to the polymorphism information content (PIC) and resolving power were estimated. All Statistical analyses were performed using the Multivariate Statistical Package “MVSP”, Version 3.21.

## 3 Results and discussion

### 3.1 ISSR and SCoT markers assay

**Figure 2:**
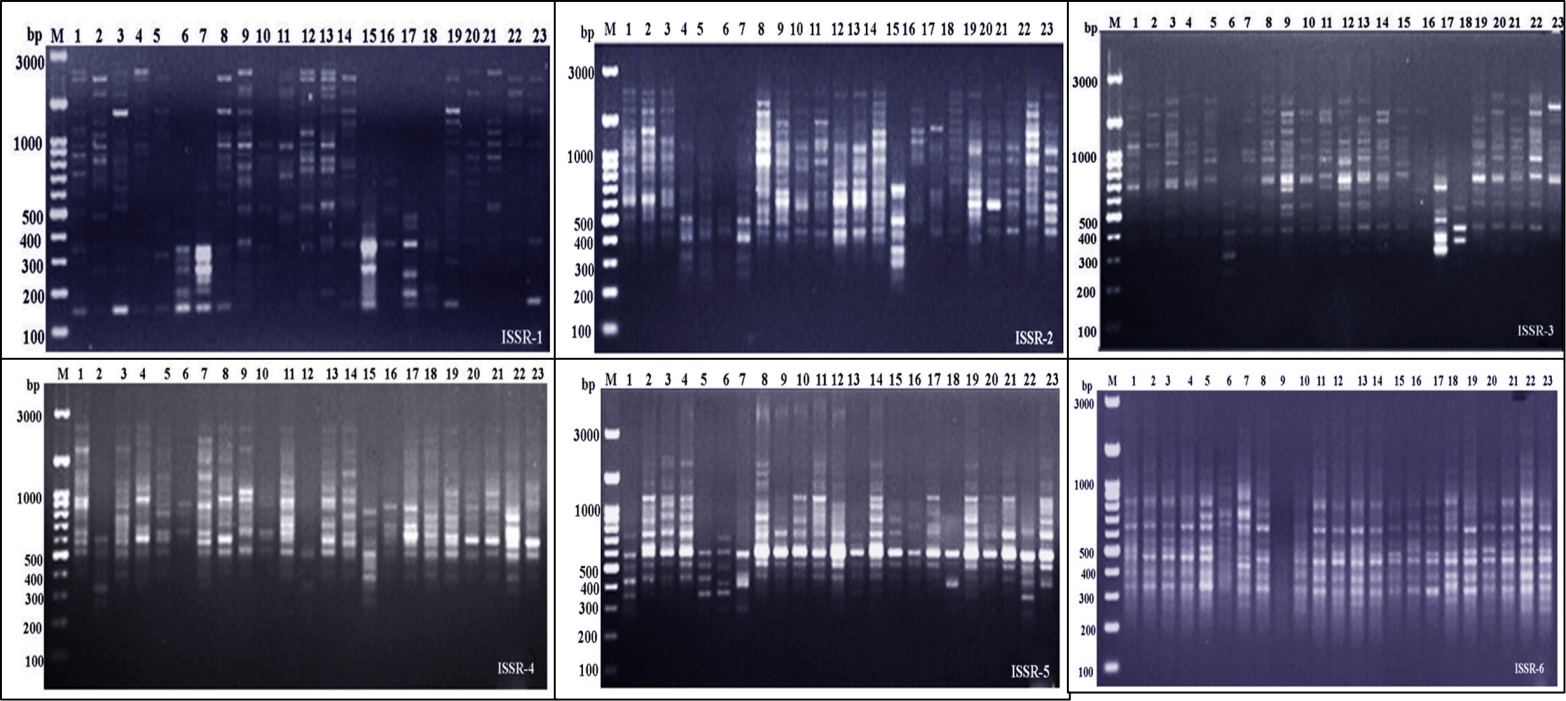
Electrophoretic profile of PCR products using ISSR primer for the 23 *Juniper* genotypes. M stands for a 100 bp marker. Lanes 1–23 correspond to all *Juniper* genotypes.

**Figure 3:**
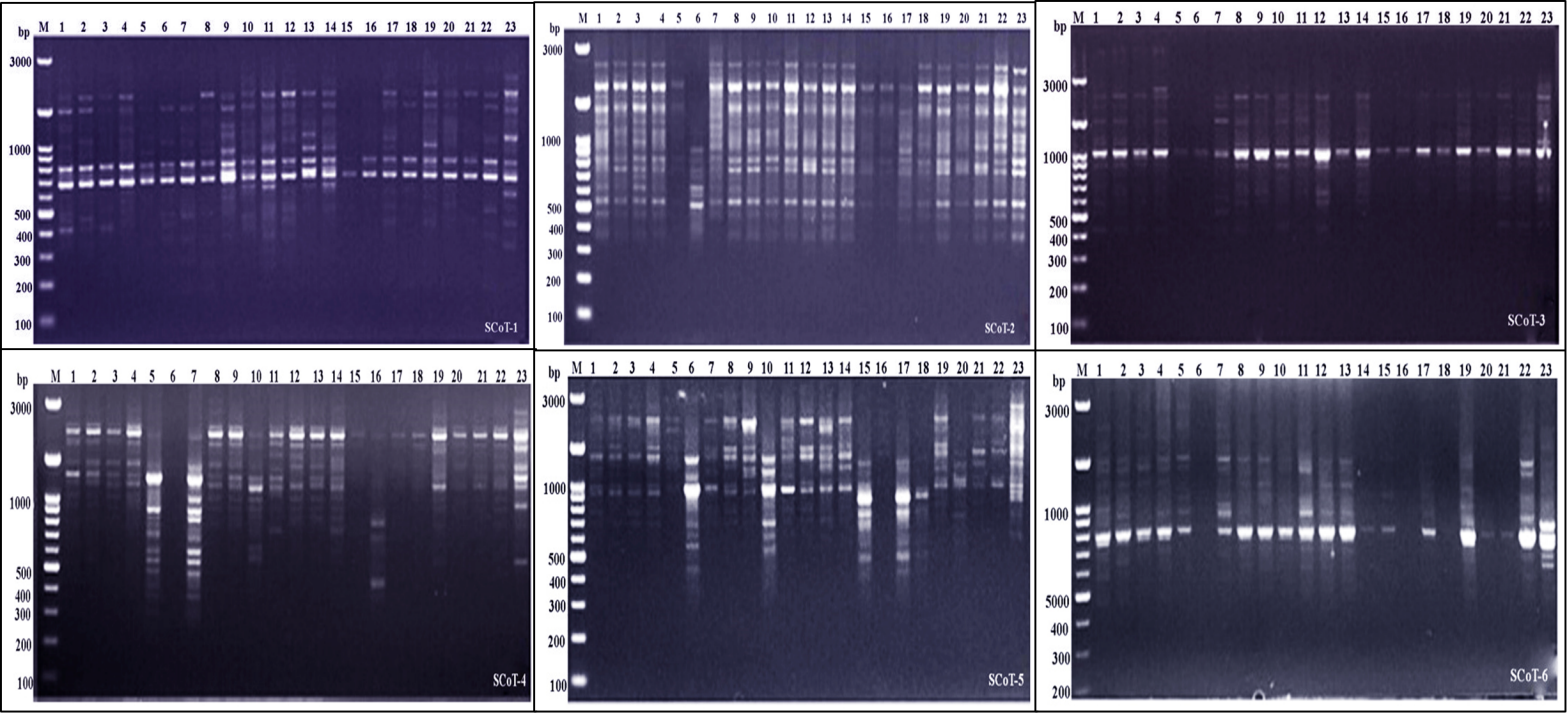
Electrophoretic profile of PCR products using SCoT primer for the 23 *Juniper* genotypes. M stands for a 100 bp marker. Lanes 1–23 correspond to all Juniper genotypes.

Molecular markers, such as the PCR-based dominant markers ISSR and SCoT, are valuable tools for characterizing genetic materials in plant breeding; these methods are highly polymorphic across various species, require minimal template DNA information, and can be analyzed without radioactivity (Shaban et al., 2022). ISSR and SCoT analyses were employed to compare the genetic makeup of 23 *J. procera* genotypes. All primers produced consistent PCR results with unique patterns for each genotype, generating informative and easy-to-interpret profiles. Six ISSR and SCoT primers were used to examine the similarities and relationships between the twenty-three genotypes (**Figures 2**, **3**). With an average of 17.2 and 17.5 bands/primer, 103 and 105 bands were amplified (**Tables 2**, **3**). The ISSR-4 and SCoT-6 produced the most bands (20 and 22), while the least number of bands (13 and 12) was recorded at the ISSR-3 and SCoT-2, respectively. However, the highest polymorphism (39% and 33%) was recorded at ISSR-1 and SCoT-5, respectively. Conversely, 22% and 17% of the lowest polymorphism values were obtained at ISSR-6 SCoT-6 primers, respectively. However, Ghorbanzadeh et al. (2021) noted concurrently with the current study that the high degree of polymorphism in the genotypes under study and the high number of scorable DNA fragments both highlight the remarkable efficacy of ISSR markers in assessing the genetic diversity of the juniper populations. Comparably, there are more DNA fragments than those reported by Khoshhal Sarmast et al. (2018), who found that 285 polymorphic DNA fragments were produced using four primers across eight populations. According to Adawy et al. (2014), ISSR is also highly polymorphic and essential for research on developmental genetics, genome mapping, and biodiversity. Also, Xiong et al. (2011) reported that ISSR is expected to be associated with comparable essential genes and traits; aside from these markers, they are multilocus, facilitating the acquisition of high hereditary polymorphism. Moreover, the average polymorphs were 27.8 and 24.3% for ISSR-1, ISSR-4, SCoT-5, and SCoT-2, respectively, with frequencies between 0.70 and 0.83 and 0.70 and 0.83. It is nearly similar to the results of Khoshhal Sarmast et al. (2018) and Ghorbanzadeh et al. (2021), who reported more DNA fragments compared to our study; the variations across studies in the number of bands and level of polymorphism may be attributed to variations in the number of individuals sampled, the type of ISSR primer utilized, and the plant species studied.

**Table 2:** Amplicons resulted from ISSR markers in *Juniper* genotypes.

No.	Primer Name	Size	TNB	PBN	P%	F	R	PIC
**1**	ISSR-1	150-2900	18	7	39	0.70	15.7	0.26
**2**	ISSR-2	230-2800	18	5	28	0.74	15.9	0.28
**3**	ISSR-3	270-2400	20	6	30	0.71	18.3	0.28
**4**	ISSR-4	280-2850	13	3	23	0.83	13.5	0.24
**5**	ISSR-5	285-2650	16	4	25	0.77	17.0	0.27
**6**	ISSR-6	230-1890	18	4	22	0.80	16.0	0.25
**Total**		–	103	29	28.1	–	–	–
**Mean**			17.2	4.8	27.8	0.76	16.1	0.26

TBN, total band number; PBN, polymorphic band number; P%, polymorphism percentage; F, frequency; R, resolving power, PIC, polymorphism information content.

**Table 3:** Amplicons resulted from SCoT markers in *Juniper* genotypes.

No.	Primer Name	Size	TNB	PBN	P%	F	R	PIC
**1**	SCoT-1	260-2500	18	5	28	0.76	17.6	0.27
**2**	SCoT-2	350-2800	22	4	18	0.85	13.3	0.23
**3**	SCoT-3	410-2900	14	3	21	0.73	15.4	0.27
**4**	SCoT-4	260-2950	21	6	29	0.75	18.1	0.27
**5**	SCoT-5	400-2900	18	6	33	0.69	18.2	0.27
**6**	SCoT-6	600-2800	12	2	17	0.72	12.3	0.27
**Total**		–	105	26	24.7	–	–	–
**Mean**			17.5	4.3	24.3	0.75	15.8	0.26

The PIC is a valuable tool for analyzing molecular data in cultivar-specific marker identification, genetic fingerprinting, accurate hybrid selection, and genetic diversity studies; its values reflect genetic diversity depending on the marker system employed (Parthiban et al., 2018). Data in **Table 3** shows the values ranging from 0.24 to 0.28 for ISSR and 0.23 to 0.27 for SCoT markers. Concurrent with the present study, Rashidi et al. (2013) reported that values between 0 and 0.2 designate low genetic variability, while values between 0.5 and 1 indicate significant observed variability. Similar to our results, Ghorbanzadeh et al. (2021) reported that the PIC fluctuated between 0.26 and 0.47. Furthermore, Yermagambetova et al. (2022) investigated *Juniperus* species from central Asia and found the PIC value varied between 0.077 and 0.662, with an average of 0.43. The genetic variation between Juniperus species is significantly influenced by the geographical region, the primer employed, and the plant species.

### 3.2 Juniper genotypes identification

Data in (**Tables 3** and **4**) illuminated unique genotype-specific ISSR and SCoT markers among the differentiated 23 Juniper genotypes. These markers serve as valuable tools for genotype-specific identification (Ghorbanzadeh et al., 2021). ISSR primers generated 60 unique markers (58.2%), represented by 36 positive and 24 negative unique bands. Juniper genotype 20 produced the highest number of unique markers, reaching nine. Conversely, four Juniper genotypes (9, 15, 19, and 21) exhibited the lowest number of unique markers, with only one band each. In parallel, SCoT markers generated 60 unique markers (57.1%), represented by 29 positive and 31 negative unique bands. The highest number of SCoT unique bands (7 bands) was recorded in *Juniper.* genotype 22, while the lowest number of unique markers was recorded in Juniper genotypes 5, 13, 19, and 23 (**Table 5**). Attractively, using two distinct markers in this study successfully identified several genotype-specific molecular markers, which allow for the differentiation of the examined Juniper genotypes and can be considered practical tools for sustainable fingerprinting and diagnostic indicators (Khoshhal Sarmast et al., 2018 and Ghorbanzadeh et al., 2021). In this way, using two distinct markers in this study successfully identified several genotype-specific molecular markers, which allow for the differentiation of the examined Juniper genotypes and can serve as molecular indicators of economic traits. These methods can be considered practical tools for sustainable fingerprinting and diagnostic indicators.

**Table 4:** Specific unique ISSR positive markers of *Juniper* genotypes.

Juniper genotypes	Primers	Bands	No. of markers	M.W (bp)
2	ISSR-03	2	ISSR-03-12	960
			ISSR-03-16	692
4	ISSR-01	2	ISSR-01-11	840
			ISSR-01-18	442
6	ISSR-03	2	ISSR-03-14	904
			ISSR-03-25	251
7	ISSR-02	1	ISSR-02-13	696
9	ISSR-03	1	ISSR-03-06	1646
15	ISSR-04	1	ISSR-04-17	429
17	ISSR-02	1	ISSR-02-05	1628
19	ISSR-02	1	ISSR-02-17	524
20	ISSR-06	1	ISSR-06-02	1553
22	ISSR-03	1	ISSR-03-10	1261
23	ISSR-06	1	ISSR-06-08	883
**Total**	**5**	**14**	**14**	**….**

No negative unique markers were found.

**Table 5:** Specific unique SCoT positive markers of *Juniper* genotypes.

Juniper genotypes	Primers	Bands	No. of markers	M.W (bp)
6	SCoT-02	1	SCoT-02-16	539
7	SCoT-02	3	SCoT-02-02	2586
	SCoT-03		SCoT-03-15	523
	SCoT-04		SCoT-04-16	634
12	SCoT-01	1	SCoT-01-07	1279
14	SCoT-01	2	SCoT-01-08	1219
	SCoT-03		SCoT-03-04	2217
15	SCoT-06	1	SCoT-06-13	1343
16	SCoT-04	1	SCoT-04-15	646
19	SCoT-03	1	SCoT-03-07	1519
23	SCoT-01	2	SCoT-01-01	2999
	SCoT-05		SCoT-05-12	1236
**Total**	**6**	**12**	**12**	**….**

### 3.3 Grouping and comparison of genotypes using ISSR and SCoT markers

**Figure 4:**
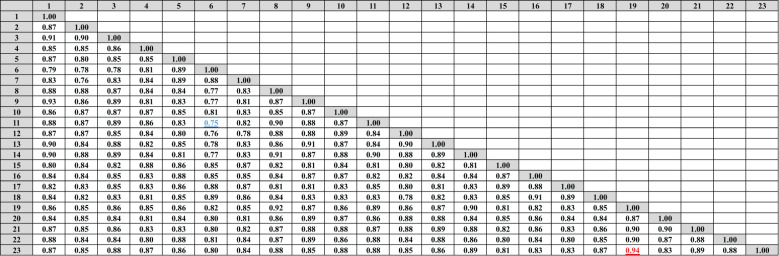
Compares the 23 cultivars of *J. procera* according to the coefficient of Dice as revealed by ISSR markers.

**Figure 5:**
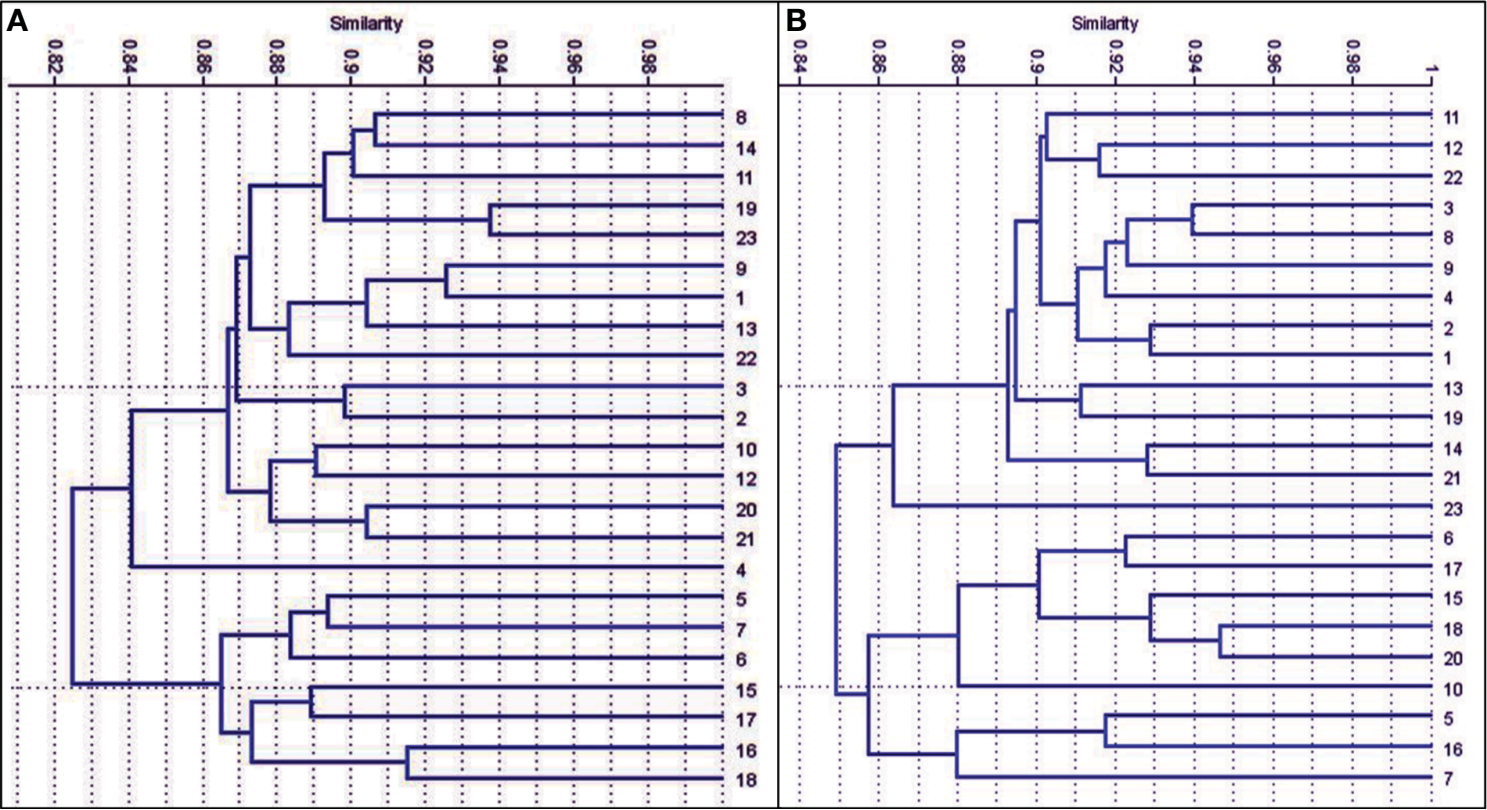
The dendrograms of 23 *J*. *procera* cultivars that were produced from the data of ISSR **(A)** and SCoT markers **(B)** using UPGMA and a similarity matrix using the Dice coefficient.

**Figure 6:**
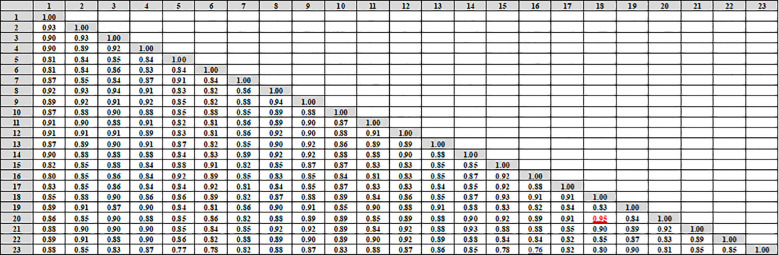
Compares the 23 cultivars of *J*. *procera* according to the coefficient of Dice as revealed by SCoT markers.

Data in (**Figures 4**, **5A**) showed that the genetic similarity fluctuated between 0.75 and 0.94, indicating a high degree of genetic similarity. Genotypes 19 and 23 showed the highest genetic similarity (0.94), followed by a similarity of 0.93 between genotypes 1 and 9, and between genotypes 6 and 11, the lowest genetic similarity (0.75) was observed. The dendrogram displayed two independent groups; the first group included seven genotypes (5, 7, 6, 15, 17, 16, and 18). The second leading group had sixteen genotypes (8, 14, 11, 9, 23, 9, 1, 13, 22, 3, 2, 10, 12, 20, 21, and 4). Regarding SCoT markers, the genetic similarity values show significant genetic relatedness between the genotypes, ranging from 0.76 to 0.95. Genotypes 18 and 20 exhibited the highest genetic similarity (0.95), followed by genotypes 3 and 8 (0.94), and the lowest genetic similarity (0.76) at genotypes 16 and 23. The dendrogram showed two core clusters; the first one has grouped nine genotypes (7, 16, 5, 10, 20, 18, 15, 17, and 16). The second one has fourteen genotypes (23, 21, 14, 19, 13, 1, 2, 4, 9, 8, 3, 22, 12, and 11) (**Figures 5B**, **6**). Because both markers could replicate different regions of the genome, they produced encouraging results and clustering in the current study (Gajera et al., 2010). As a result, according to Ghorbanzadeh et al. (2021), these markers offer more comprehensive and varied information about the genetic diversity of *Juniperus procera* accessions and within them. In several cases, dendrograms produced by different markers have made inconsistent results, as seen in the cases of snake melons, sponge gourds, and bamboo (Shaban et al., 2022).

### 3.4 Principal coordinate analysis

**Figure 7:**
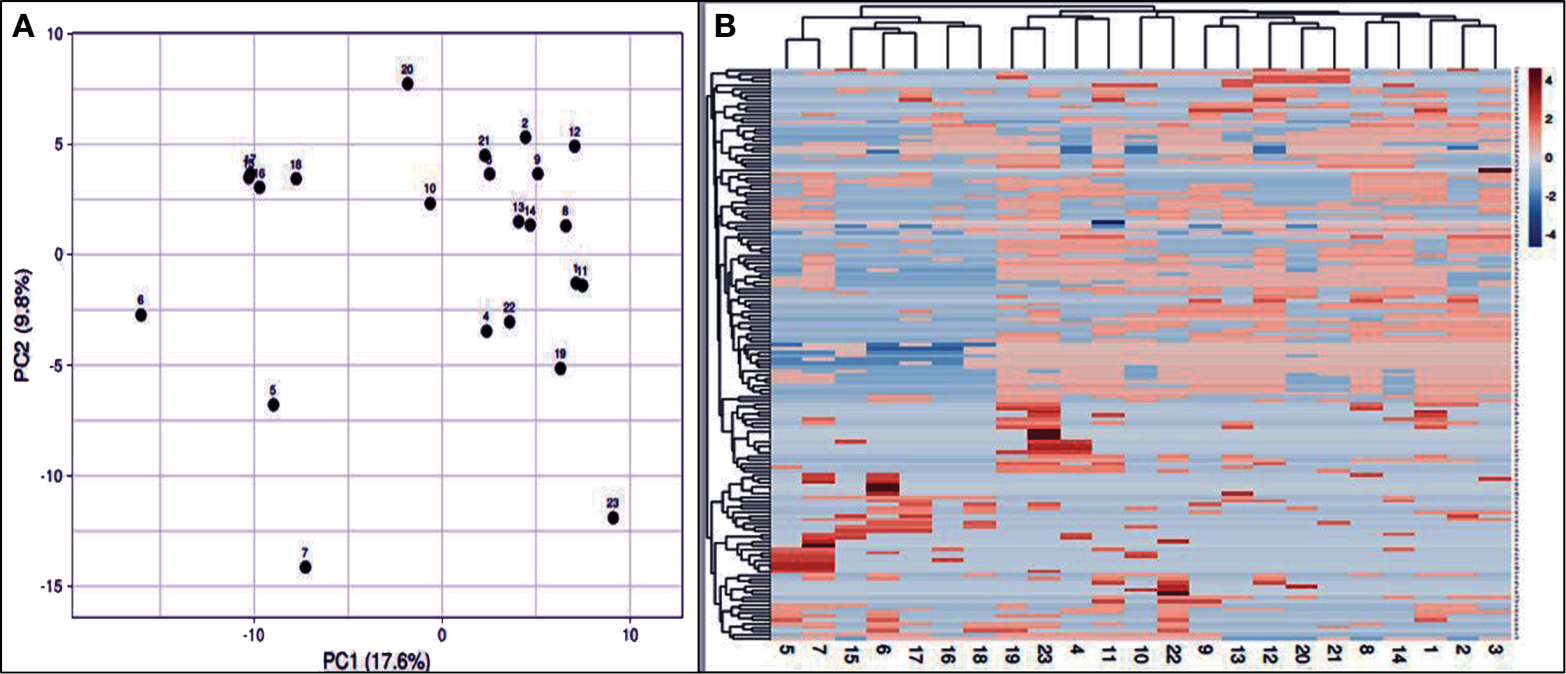
PCA based on the calculation of the first three coordinates according to the data analysis of the twenty-three *J*. *procera* genotypes **(A)**. Heat map analysis of the 23 *J*. *procera* genotypes **(B)**. Blue indicates low intensity, and red indicates high intensity.

Genetic relationships among the 23 genotypes were evaluated by PCA (**Figure 7A**). This multivariate method was used to support the grouping results obtained from the preliminary cluster analysis, which showed better resolution for closely related populations. The bunching analysis and the PCA evaluation of the structure were agreed upon. Rows are subjected to unit variance scaling, and SVD with imputation is employed to determine the major components. The X and Y axes show principal components 1 and 2, representing 17.6% and 9.8% of the overall variance. N = 23 data points. The heat map was created based on ISSR, and SCoT results to a considerable extent in the 23 *J. procera* genotypes (**Figure 7B**). Due to the differences in plant species and the lack of PCA calculation in the previous studies, it was not possible to draw meaningful comparisons or directly incorporate their findings into our dendrogram analysis. Geological and climatic variations impact the Juniperus species genome’s genetic structure significantly. Co-dominant markers appear as DNA bands containing various alleles on a gel. In contrast, dominant markers only have two alleles signified as present or absent bands and are defined by the differences observed in bands on electrophoretic gels (Alotaibi and Abd-Elgawad, 2022). Co-dominant polymorphic markers provide a more detailed picture of genetic variation by detecting all three possible genotypes (homozygous dominant, homozygous recessive, and heterozygous) (Ghorbanzadeh et al., 2021; Shaban et al., 2022).

In comparison, dominant markers only detect two genotypes (homozygous dominant and heterozygous/recessive) (Amiteye, 2021). Therefore, co-dominant polymorphic markers offer a finer-grained view of genetic diversity by revealing all possible genotype combinations. In contrast, dominant markers only indicate the presence or absence of a particular allele.

## 4 Conclusion

Genetic variations exist among the 23 *J. procera* genotypes cultivated in Saudi Arabia, as revealed by ISSR and SCoT marker analyses. Both marker investigations categorized these cultivars into two distinct genetically diverse groups, labeled as Groups 1 and 2. Additionally, specific cultivars were identified within regional sub-clusters. Thus, these findings demonstrated the utility of ISSR and SCoT markers in elucidating the genetic relationships between *J. procera* populations, which will prove valuable for sustainable *J. procera* breeding in Saudi Arabia in the future.

## Data availability statement

The original contributions presented in the study are included in the article/supplementary material. Further inquiries can be directed to the corresponding author.

## Author contributions

HA: Writing – review & editing, Writing – original draft, Visualization, Validation, Supervision, Investigation, Conceptualization. RA: Writing – review & editing, Writing – original draft, Visualization, Methodology, Investigation, Data curation, Conceptualization.
